# Adherence to dietary approaches to stop hypertension (DASH) diet in relation to psychological function in recovered COVID-19 patients: a case–control study

**DOI:** 10.1186/s40795-022-00633-5

**Published:** 2022-11-11

**Authors:** Zahra Khorasanchi, Asieh Ahmadihoseini, Omalbanin Hajhoseini, Reza Zare-Feyzabadi, Masoumeh Haghighi, Masoumeh Heidari, Ali Jafarzadeh Esfehani, Zahra Dehnavi, Payam Sharifan, Simin Rashidianyazd, MohammadReza Latifi, Fatemeh Rastgooy, Danial Ildarabadi, Maryam Mohammadi Bajgiran, Sara Saffar Soflaei, Gordon Ferns, Majid Ghayour Mobarhan

**Affiliations:** 1 Department of Nutrition, School of Medicine, Mashhad University of Medical Sciences, Mashhad, Iran; 2 Student Research Committee, Faculty of Medicine, Mashhad University of Medical Sciences, Mashhad, Iran; 3 Department of Nutrition, Ghaem Hospital, Mashhad University of Medical Sciences, Mashhad, Iran; 4 Department of Nutrition, International UNESCO Center for Health Related Basic Sciences and Human Nutrition, Faculty of Medicine, Mashhad University of Medical Sciences, 99199-91766 Mashhad, Iran; 5 Metabolic Research Center, Mashhad University of Medical Sciences, Mashhad, Iran; 6 Department of Biology, Mashhad Branch, Islamic Azad university, Mashhad, Iran; 7 Nutrition Sciences, Varastegan Institute for Medical Sciences, Mashhad, Iran; 8 Brighton and Sussex Medical School, Division of Medical Education, Brighton, UK

**Keywords:** DASH diet, COVID-19, Depression, Anxiety, Stress

## Abstract

**Background:**

Follow-up of patients after recovery from coronavirus disease 2019 (COVID-19) and identifying the adverse effects of the disease in other organs is necessary. Psychiatric symptoms can persist after patients recover from the infection.

**Aim:**

We aimed to examine the adherence to the dietary approach to stop hypertension (DASH) diet in relation to psychological function in individuals who have recovered from COVID-19.

**Method:**

This case–control study was conducted on 246 eligible adults (123 cases and 123 controls). A valid and reliable food frequency questionnaire (FFQ) was used to determine dietary intake. Depression, anxiety and stress, insomnia, sleep quality, and quality of life of participants were evaluated using DASS, Insomnia Severity Index (ISI), Pittsburgh Sleep Quality Index (PSQI), and SF-36 questionnaires, respectively.

**Results:**

There was a significant inverse correlation between total depression score with vegetables, depression, anxiety, and stress score and dietary intake of nuts, legumes, and whole grains (*p* < 0.05). There was a significant positive correlation between stress scores and the intake of red and processed meat (*P* < 0.05). In multivariate-adjusted regression model, a significant association was found between adherence to DASH diet and depression and stress only in case group (OR = 0.7863, 95% CI 0.746–0.997, *p* = 0.046 and OR = 0.876, 95% CI 0.771–0.995, *p* = 0.042, respectively).

**Conclusion:**

Adherence to a DASH diet might be associated with depression and stress reduction in recovered COVID-19 patients.

## Introduction

Severe Acute Respiratory Syndrome Coronavirus 2 (SARS-CoV-2) appears to have originated in Wuhan, Hubei Province, China, in December 2019 and resulted in a disease that was later called Coronavirus Disease 2019 (COVID-19) by the World Health Organization (WHO) [1]. SARS-CoV-2 infection may cause severe systemic disease that affects several organ systems, particularly the lungs. COVID-19 may be accompanied with long term conditions even after recovery (Long COVID). Hence, it is necessary to follow up on patients suffering from COVID-19 after recovery and identify the adverse effects of the disease in other organs [2].

Recovered covid-19 patients describe diverse symptoms such as fatigue, fever, and cough after hospitalization. Due to multi-organ impairments caused by the infection, possible psychological damage also needs to be considered [2]. It has been reported that COVID-19 patients experienced cognitive deficits after infection, including memory loss, loss of concentration, insomnia, depression, and anxiety [2]. These symptoms can persist after recovery from COVID-19. Impaired mental health affects the quality of life (QoL) [3]. Therefore, psychological impairment is an important concern in COVID-19 patients and should be considered during hospitalization and recovery. Based on a recent study, 35% of COVID-19 patients had moderate to severe psychological symptoms [4]. Likewise, in another research, 19% of COVID-19 patients had clinically significant symptoms of depression and 10.4% had mild to severe symptoms of anxiety after hospital discharge [5]. Khademi et al. revealed that the prevalence of anxiety and depression in recovered COVID-19 patients was 5.8% and 5.0%, respectively [6].

The Dietary Approaches to Stop Hypertension (DASH) diet was developed to reduce the prevalence of hypertension [7]. DASH diet is characterized by high intake of vegetables, whole grains, fruits, nuts, and leguminous and low consumption of red meat, low-fat dairy, desserts, sugary beverages and sweets [7]. Several investigations have shown that the DASH diet has beneficial effects on cardiovascular and renal disease [8, 9]. Few studies have been performed to assess the effects of the DASH diet on mood disorders [10]. Nevertheless, two studies have indicated that DASH diet improves depression score [11, 12]. Also, one investigation has shown that DASH diet improves mood disorders in women after menopause [13]. The relationship between dietary patterns and COVID-19 outcomes have been evaluated in previous studies but to the best of our knowledge, this relationship has not yet been evaluated among long COVID patients [14–17]. Psychological impairments are crucial concerns during hospitalization and recovery in COVID-19 patients. In this study, we aimed to investigate the relationship between compliance with DASH diet and psychological function in recovered covid-19 patients.

## Methods

### Study design

The current case–control study was conducted on adults aged ≥ 30 years who referred to the clinic of Qaem Hospital, Mashhad, Iran, from November 2020 to January 2021. All cases of COVID-19 in the past 1 month who had negative PCR test or CT scan at the time of interview were included in the study as case group. The exclusion criteria for case group were: receiving anti-depressant treatment during the previous six months, hepatic or renal failure, autoimmune diseases, cancer, metabolic bone disease, and having special dietary habits, such as vegetarian diet.

The control group was then selected randomly among adults > 30 years without a history of COVID-19 who were referred to the nutrition clinic of the Qaem Hospital. The exclusion criteria for controls were history of COVID-19 based on PCR or CT scan, receiving anti-depressant treatment during the previous six months, metabolic bone disease, hepatic or renal failure, autoimmune diseases, cancer, and having special dietary habits. One control was enrolled for each case and the case and control groups were matched based on gender and age (± 5 years). Due to the difficulties in recruiting controls during the study period (first and second peaks of COVID-19), and considering the consecutive patient requitement, controls were only matched based on age and gender with the case group. Furthermore, researchers were instructed to reduce the interview time in order to reduce the duration of possible COVID-19 exposure in the clinic. Therefore, only main demographic data including age, gender, and education level were collected in the study.

Of the 246 eligible individuals who participated in the present study, a total of 240 participants (120 controls and 120 cases) were included in the final analysis. Two cases and four controls were excluded from the study because their mean energy intakes were outside ± 3 SD. All participants provided informed written consent, and all methods were carried out in accordance with relevant guidelines and regulations or the declaration of Helsinki.

### General and anthropometric characteristics

Anthropometric and demographic characteristics, including age, education level, weight, and height, were collected by a trained nurse. A calibrated personal scale was used for weight measurement. To determine height, fixed measuring tape on the wall was used. The following formula was applied for calculating body mass index: body weight (kg)/(body height (m))^2^.

### Dietary assessment

A 68-item food frequency questionnaire (FFQ) was used to evaluate the food intake of subjects. The validity and reliability of the FFQ has been approved in a previous study [18]. The FFQ was filled during face-to-face interviews. To calculate nutrient and energy intakes, the portion sizes in FFQ were converted to grams and were analyzed by nutritionist IV software (N-Squared Computing, Cincinnati, OH, USA). The intake of the nutrients was adjusted based on energy intake as intakes/ 1000 kcal. A DASH score was calculated based on the method proposed by Fung et al. [19]. The components of DASH score consisted of 8 items including Fruit, Vegetable, Nuts and legumes Whole grains, Red and processed meat, Low fat dairy product, Sweetened beverage and Sodium. DASH score provides a scale to identify high intake of vegetables, legumes and nuts, fruits, whole grains and low-fat dairies based on quintile classification (highest quintile is considered as 5 points and lowest quintile is considered as 1 point). Individuals with lower quintile of sodium intake, red and processed meats as well as sweetened beverages receive higher points. Eventually, the total component scores were combined to obtain the general DASH score that ranged between 8 and 40 points.

### Depression anxiety stress scales (DASS)

The depression anxiety stress scales (DASS) is a questionnaire on mood status [20]. DASS-21 is an accurate and valid tool that consists of 21 items. Each question is rated on a four-point Likert scale (0–3) to recognize the severity of stress, anxiety, and depression. The score of each sub-class should be doubled in order to be comparable with the scores of the main questionnaire that is composed of 42 items (DASS-42). Lower DASS scores demonstrate lower degrees of negative emotions. The reliability and validity of DASS have been previously reported in the Iranian population [21]. The scores for stress, anxiety, and depression were allocated into two categories: No or minimal disorder; and some degree of mood disorder. The scores obtained from each subscale were determined as follows: in depression subscale, scores ≤ 9 indicated no depression and scores higher than 9 indicated some degree of depression, in anxiety subscale scores ≤ 7 indicated no anxiety and scores higher than 7 indicated some degree of anxiety, and in stress subscale scores ≤ 14 indicated no anxiety and scores higher than 14 indicated some degree of stress.

### Insomnia Severity Index (ISI)

The insomnia severity index (ISI) is a seven-item self-report questionnaire for assessing insomnia symptom and their consequences. ISI dimensions include anxiety-associated sleeping disturbance, satisfaction with the present sleep pattern, and sleep disorder intensity [22]. Each item is scored on a scale of 0 to 4. The overall ISI score ranges from 0 to 28. Categories of insomnia are defined based on ISI scores as follows: no insomnia (0–7) and mild to severe insomnia (8–28). In this study, we used the validated and reliable Persian version of ISI for the Iranian population (Cronbach's alpha > 0.8 and intra-class correlation coefficient > 0.7) [23].

### Pittsburgh Sleep Quality Index (PSQI)

PSQI questionnaire is a 19-item self-reported tool to evaluate sleep quality over the past 30 days [24]. This questionnaire contains 7 component scores, including daytime dysfunction, use of sleep medication, sleep disturbances, habitual sleep efficiency, subjective sleep quality, sleep duration, and sleep latency. The answers are scored on a 3-point scale (0–3). Therefore, the total PSQI score ranges from 0 to 21. Patients were categorized into two groups according to their PSQI score: the poor-sleeper group (PSQI > 5) and the good-sleeper group (PSQI ≤ 5). Farrahi Moghaddam et al. validated the Persian version of PSQI in 2012 [25].

### Quality of Life Questionnaire

The Short Form Health Survey (SF-36) was used as a reliable tool for assessing the general quality of life. Scores on this questionnaire range from 0 to 100. validity and reliability of the Persian version of SF-36 were evaluated in a previous study [26].

### Statistical analysis

Statistical analysis was run by SPSS Statistics for Windows v20 (SPSS, Inc., Chicago, IL). The Kolmogorov–Smirnov test was used to analysis the normality of variables. Normally distributed variables were expressed as mean and standard deviation (SD), and non-normally distributed variables were expressed as the median and interquartile range (IQR). Also, frequency and percentage were demonstrated for categorical variables. Chi-square test and independent sample t-test were applied for comparing variables between case and control groups, respectively. To compare dietary intakes between case and control as well as tertiles of DASH diet, a Multivariate Analysis of Variance (MANOVA) test was used. Finally, multinomial logistic regression was performed to evaluate the relationship between tertiles of DASH diet and DASS-21 subscale scores. Various models were designed with study variables as confounders. The best model based on model fit, power and sensitivity analysis was presented in the results. For all analyses *p* value of < , 0.05 was considered to be statistically significant.

## Results

### Demographic and anthropometric characteristics of the population

Anthropometric and demographic parameters of the subjects in case and control groups are presented in Table 1. The mean ± SD age of the case group was 60.38 ± 13.61 years, of which 57.5% were male. There were no significant differences in age, gender, weight, and BMI between the two groups (*p* > 0.05). However, there was a significant difference in height and educational level between groups (*p* < 0.05).

**Table 1:** Demographic and clinical characteristics of the participants between groups

**Variables**	**Case (*n* = 120)**	**Control (***n*** = 120)**	***P*-value** ^**a**^
**Gender**			
Male	69 (57.5%)	65 (54.2%)	0.512
Female	51 (42.5%)	55 (45.8%)	
**Age (year)**	60.38 ± 13.61	57.43 ± 7.71	0.321
**Educational level**			
Illiterate and High school	46 (38.4%)	20 (16.7%)	** < 0.001**
Diploma	61 (50.8%)	36 (30%)	
Bachelor and higher	13 (10.8%)	64 (53.3%)	
**Anthropometric**			
Height (cm)	168.92 ± 9.08	165.21 ± 9.45	**0.004**
Weight (kg)	77.52 ± 18.40	76.78 ± 13.40	0.731
BMI (kg/m^2^)	26.99 ± 6.15	28.06 ± 3.92	0.151

*BMI* Body Mass Index

Data presented as Mean ± SD or n (%)

^a^Obtained from *t* test for continuous variables and χ2 test for categorical variables

### Comparison of diet and psychological function between groups

Table 2 shows the comparison of the mean dietary intake of subjects in tertiles of adherence to the DASH-diet style between case and control groups. There was a significant difference in protein and vegetable intakes between case and control groups (*p* = 0.003, 0.016, respectively). Regarding components of DASH diet, there were significant differences between intakes of nuts and legumes, fruit and whole grain, and tertiles of DASH diet between groups (*p* < 0.05). There was a significant difference in red and processed meat, low-fat dairy product, and protein intakes between tertiles of DASH diet (*p* < 0.05).

**Table 2:** Energy-adjusted of dietary intakes between groups

**Variable**	**Case (*n* = 120)**			**Control (*n* = 120)**			***P*-value **^**#**^
	**T1**	**T2**	**T3**	**T1**	**T2**	**T3**	
^**a**^**Dietary Macronutrient intake (g/day)**							
Carbohydrate	199.15 ± 100.42	223.81 ± 71.69	238.22 ± 91.95	239.56 ± 50.15	266.23 ± 68.53	275.24 ± 87.97	0.982
Protein	89.52 ± 29.08	73.17 ± 20.59	72.72 ± 21.87*	78.52 ± 15.20	83.67 ± 10.57	76.81 ± 16.27	**0.003**
Fat	109.92 ± 44.27	111.08 ± 26.27	106.67 ± 34.52	100.84 ± 18.76	89.63 ± 25.89	90.18 ± 30.45	0.464
^**a**^**Components of DASH (g/day)**							
Fruit	214.12 (69.76–359.12)	293.30 (152.81–527.78)	530.88 (356.40–763.69)**	217.50 (169.13–314.90)	299.41 (216.80–416.18)	448.68 (348.49–608-10)**	0.642
Vegetable	108.04 (63.14–203.71)	72.24 (21.55–126.0)	172.01 (95.50–241.40)	72.48 (45.19–118.90)	118.60 (74.07–163.80)	119.18 (57.13–172.83)	**0.016**
Nuts and legumes	7.57 (0.04–17.38)	12.02 (3.87–18.31)	23.97 (13.41–38.23)*	17.43 (9.50–29.74)	25.86 (18.78–45.23)	30.58 (24.14–52.0)*	0.487
Whole grains	28.54 (6.38–66.56)	108.54 (71.85–181.74)	96.10 (35.94–138.44)*	97.20 (48.42–179.95)	165.80 (117.50–258.29)	155.35 (85.03–264.68)**	0.754
Red and processed meat	53.61 (29.74–96.85)	32.31 (21.28–63.09)	27.57 (13.83–51.55)*	55.0 (40.90–78.05)	50.10 (28.76–71.15)	37.45 (25.91–58.73)	0.844
Low fat dairy product	182.60 (58.66–376.18)	120.79 (27.74–273.31)	49.97 (32.75–98.36)*	146.41 (77.99–275.91)	121.94 (51.85–206.82)	73.87 (25.45–101.20)	0.083
Sweetened beverage	53.61 (29.74–96.85)	32.31 (21.28–63.09)	27.57 (13.83–51.55)	60.08 (37.17–132.99)	42.86 (19.66–79.70)	18.72 (4.55–52.63)*	0.221
Sodium	1551.72 ± 680.52	1432.51 ± 482.90	1368.90 ± 605.39	1792.08 ± 442.61	1904.82 ± 634.39	1685.74 ± 869.69	0.536

Obtained from Multivariate analysis of variance (MANOVA) test

Data presented as Mean ± SD or Median (IQR)

^*^p < 0.05 and **p < 0.001 within tertiles of DASH

^#^*p*-value for differences between case and control group

^a^The parameters are adjusted based on the energy intakes

**Fig. 1:**
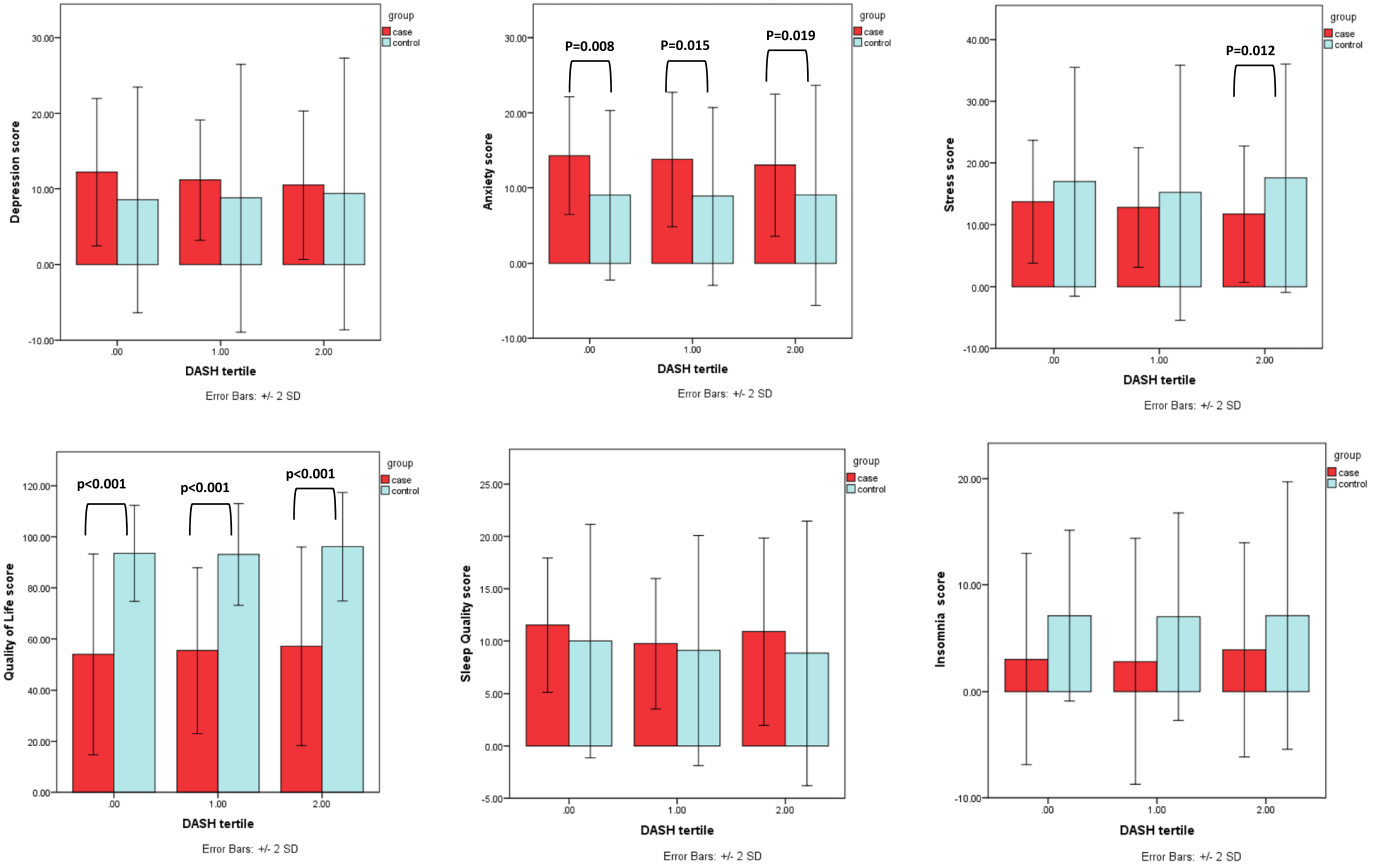
Comparison between DASH tertiles and psychological faction in case and control group

Comparison of psychological scores and tertile of DASH diet between case and control groups is presented in Fig. 1. There was a significant difference in QoL of first, second and third tertiles of DASH between case and control groups (*p* < 0.001). Also, there was a significant difference in anxiety in first, second and third tertiles of DASH between the case and control groups (*p* = 0.008, *p* = 0.015, *p* = 0.019, respectively). There was a significant difference in the stress of third tertiles of DASH between the case and control groups (*p* = 0.012).

As observed in Table 3, There was a significant inverse correlation between total depression scores and the intake of vegetables (*r* =  − 0.290; *P* < 0.05) and between depression, anxiety, and stress scores and the intake of nuts, legumes (*r* =  − 0.224; *P* < 0.05, *r* =  − 0.280; *P* < 0.05, *r* =  − 0.237; *P* < 0.05 respectively) and whole grains (*r* =  − 0.224; *P* < 0.05, *r* =  − 0.359; *P* < 0.05, *r* =  − 0.283; *P* < 0.05 respectively). A significant positive correlation was seen between stress and the intake of red and processed meat (*r* = 0.231; *P* < 0.05).

**Table 3:** Correlation coefficient between psychological tests and components of DASH diet in case and control group

**Food groups (g/day)**^a^	**depression**		**anxiety**		**stress**		**Insomnia**		**Sleep Quality**		**Quality of life**	
	**case**	**control**	**case**	**control**	**case**	**control**	**case**	**control**	**case**	**control**	**case**	**control**
	**r**	**r**	**r**	**r**	**r**	**r**	**r**	**r**	**r**	**r**	**r**	**r**
Fruit	-0.193	0.109	-0.163	-0.092	-0.054	0.090	-0.113	0.042	-0.015	-0.027	0.049	0.111
Vegetable	-0.290*	-0.069	-0.138	-0.061	-0.183	-0.091	0.044	-0.025	0.004	-0.033	0.053	-0.008
Nuts and legumes	-0.224*	-0.134	-0.280*	-0.077	-0.237*	-0.006	-0.028	-0.038	0.059	-0.091	0.101	0.053
Whole grains	-0.224*	-0.114	-0.359**	-0.025	-0.283*	-0.034	-0.093	-0.072	-0.057	-0.075	0.093	0.042
Red and processed meat	0.181	-0.098	0.114	-0.067	0.231*	-0.106	-0.026	0.015	-0.070	0.023	-0.122	-0.073
Low fat dairy product	-0.135	0.008	-0.160	-0.114	-0.134	-0.062	0.025	0.023	0.017	-0.026	0.015	-0.073
Sweetened beverage	0.091	-0.037	-0.061	0.022	0.055	-0.028	0.012	0.085	-0.010	0.148	0.005	0.066
Sodium	-0.164	-0.121	-0.171	0.036	0.213	-0.011	-0.162	-0.031	0.048	0.066	0.177	0.004

^*^p < 0.05

^**^p < 0.01

^a^The parameters are adjusted based on the energy intakes

### Association between psychological function and DASH diet

In multinomial logistic regression analyses, the first tertile of DASH diet in each group was served as reference. Multivariable-adjusted odds ratios for psychological function across tertiles of DASH diet are presented in crude and adjusted models in Table 4. In the adjusted model, the odds ratio was adjusted for energy intake, age, sex, and educational levels. Only in case group, adherence to DASH diet was significantly associated with depression and stress in the adjusted model (OR = 0.7863, 95% CI 0.746–0.997, *P*-value = 0.046; OR = 0.876, 95% CI 0.771–0.995, *p*-value = 0.042, respectively).

**Table 4:** Multiple-adjusted odds ratio (OR) and 95% confidence intervals (CI) between tertiles of DASH style

	**Case (*n* = 120)**			**Control (*n* = 120)**		
	**T1**	**T2**	**T3**	**T1**	**T2**	**T3**
**Depression**						
Crude	Ref	0.956 (0.843–1.085)	0.921 (0.815–1.042)	Ref	1.004 (0.953–1.057)	1.012 (0.961–1.065)
Adjusted model	Ref	0.915 (0.796–1.052)	0.863 (0.746–0.997)*	Ref	1.003 (0.952–1.058)	0.019 (0.966–1.074)
**Anxiety**						
Crude	Ref	0.975 (0.851–1.117)	0.935 (0.823–1.063)	Ref	0.996 (0.930–1.067)	1.000 (0.933–1.072)
Adjusted model	Ref	0.934 (0.804–1.085)	0.884 (0.761–1.026)	Ref	0.997 (0.929–1.069)	1.002 (0.934–1.076)
**Stress**						
Crude	Ref	0.968 (0.863–1.084)	0.924 (0.827–1.033)	Ref	0.980 (0.937–1.025)	1.006 (0.962–1.053)
Adjusted model	Ref	0.927 (0.816–1.052)	0.876 (0.771–0.995)*	Ref	0.979 (0.935–1.026)	1.007 (0.960–1.055)
**Insomnia**						
Crude	Ref	0.991 (0.886–1.109)	1.032 (0.936–1.137)	Ref	0.996 (0.915–1.084)	1.000 (0.918–1.090)
Adjusted model	Ref	0.991 (0.880–1.116)	1.030 (0.926–1.145)	Ref	0.996 (0.913–1.085)	1.001 (0.918–1.092)
**Sleep Quality**						
Crude	Ref	0.874 (0.721–1.059)	0.959 (0.823–1.117)	Ref	0.973 (0.905–1.047)	0.965 (0.895–1.041)
Adjusted model	Ref	0.844 (0.681–1.048)	0.912 (0.758–1.098)	Ref	0.973 (0.904–1.048)	0.960 (0.888–1.038)
**Quality of Life**						
Crude	Ref	1.004 (0.975–1.034)	1.009 (0.982–1.038)	Ref	0.995 (0.954–1.039)	1.028 (0.983–1.075)
Adjusted model	Ref	1.008 (0.978–1.038)	1.014 (0.986–1.044)	Ref	0.996 (0.952–1.042)	1.031 (0.982–1.082)

Obtained from multinomial logistic regression according tertile of DASH score

Model 1: Adjusted for age, gender and energy intake and education stage

^*^p < 0.05

## Discussion

In this study, we found that high adherence to a DASH-type diet, with high consumption of vegetables, fruits, seeds, legumes, and nuts as well as low consumption of sodium, red and processed meats, was associated with lower odds of depression and stress in recovered COVID-19 patient.

Although the association between dietary styles and mental well-being is unclear, it is possible that mental health is related to dietary habits. Previous studies have recommended the intake of fruit and vegetables and whole-grains in COVID-19 [27, 28]. Nutrition institutes from Spain and Italy have recommended to include at least five servings of vegetables and fruits daily in the diet of patients with COVID-19 [27]. Vegetables and fruits contain a high amount of minerals and vitamins that are important in the modulation of the immune system. Also, vegetables and fruits contain various antioxidants, water, and fiber. All of these nutrients play an important role in the control of diabetes, hypertension, and weight gain, which increase the severity of COVID-19 [29]. As well, COVID-19 patients had mood disorders, including depression and anxiety [2]. In line with our results, Valipour et al. conducted a study on 3846 individuals using of 106-item FFQ in Isfahan province in 2010 and reported that moderate adherence to DASH-style diet was related to lower odds of depression in adult population but this relationship was not significant among obese, overweight, and male adults [11]. Also, high adherence to DASH diet was reported to be inversely correlated with anxiety in normal-weight adults in another study [7]. Faghih et al. performed a study on 240 Iranian university students using 168-item FFQ containing and reported that greater adherence to DASH dietary pattern, characterized by high intake of vegetables and fruits, nuts, seeds and legumes, low-fat dairy products, and whole grains, was significantly correlated to psychological well-being and remarkably decreased depression, anxiety, and stress scores [30]. Based on clinical evidence, gastrointestinal microbiota contribute to communication between the gastrointestinal and brain and play a role in behavior and mood disorders. In addition, the quality of diet also affects the gastrointestinal microbiota, which is associated with stress, depression, and anxiety [31].

Diet and lifestyle improvement may play an important role in the symptoms of mood disorder. We found that depression scores were inversely correlated with the intake of vegetables among recovered COVID-19 patients, which is similar to previous studies [32, 33]. A meta-analysis consisting of 18 investigations and 446,551 participants has reported that vegetable intake is inversely correlated to the risk of depression [32]. In addition, a cohort study indicated that regular and frequent consumption of vegetables and fruits may play an important role in decreasing the risk of depression in elderly persons [33]. Other studies have reported that a high intake of snacks and fatty foods and low consumption of vegetables and fruits was associated with increased severity of stress in children [34]. A systematic review reported that high consumption of vegetables might reduce depressive symptoms and promote higher levels of self-efficacy [35]. Although the mechanisms by which vegetables or healthy dietary style reduce the risk of depressive behavior is not entirely clear [33], some of the nutrients, including antioxidants, n-3 fatty acids, and B complex vitamins, possibly be associated with mood disorders [36]. Inflammation and oxidative stress can influence autonomic nervous system neurons that consequently increase depressive symptoms. B complex vitamins have a beneficial effect on oxidative stress and inflammation and are crucial for the function of neurons [37]. Therefore, deficiency in these nutrients is related to depression and anxiety [37]. Another possible mechanism for improving depressive symptoms is phytochemical ingredients in vegetables and fruits, which have antioxidant activity [33].

Results of the present study indicated that the intake of nuts, seeds, and legumes was associated with reduced prevalence of depression, anxiety, and stress scores among recovered COVID-19 patients. Our findings were in line with the results of previous investigations [38]. Nuts, seeds, and legumes contain a remarkable amount of macro- and micro-nutrients, polyphenol antioxidants, and beneficial bioactive ingredients that their deficiency leads to inflammation and oxidative stress, consequently promoting psychological distress and decreasing quality of life [39]. We found an inverse association between whole grains intake and depression, anxiety, and stress among recovered COVID-19 subjects. Similar to our results, Sangouni et al. showed that higher intake of whole grains was associated with reduced odds of depression [40]. Also, a study conducted on 3172 adult subjects with the age range of 18–55 years revealed that moderate whole grain consumption was inversely correlated with anxiety in females [41]. In contrast to our results, some studies showed no significant effect for whole grains on mood disorders [42, 43]. It has been shown that the consumption of whole grains reduces insulin resistance, which is related to psychological disorders such as anxiety and depression. Moreover, whole grains contain B-vitamins, which play an important role in neuronal function [41, 44].

Also, we found stress scores were positively correlated with the intake of red and processed meat among recovered COVID-19 patients. Some research has evaluated food choices after stressful events [45, 46]. Uemura et al. determined food group intake after the Earthquake in eastern Japan in 2011 and revealed that people with higher stress had worse diet quality, i.e., less intake of vegetables, fruits, and soy as well as a high intake of meat [46]. Also, after the spring of 2020, some studies were conducted on food choices after the COVID-19 lockdown. For instance, a Polish survey showed decreased consumption of fruits and vegetables and increased intake of dairy and meat [47]. On the other hand, earlier studies have also suggested a positive relationship between high intakes of red and processed meat and increased risk of stress [48, 49]. Overall, these results showed that dietary intakes might influence the risk of stress, and thus, future large-scale research is required to evaluate the dietary determinants of stress.

To the best of our knowledge, this study was the first study examining the correlation between psychological functions and adherence to DASH diet among recovered COVID-19 patients. Despite the strengths of this study (e.g., population-based study), some limitations should be considered. We did not measure physical activity in our subjects. Due to the movement restriction act in the country, it was hypothesized that the level of physical activity during the past one moth was affected by the restriction in outdoor activities and was similar in both the case and control groups. In our study we used a 68-item food frequency questionnaire (FFQ) to evaluate food intake and diet status of subjects. As FFQ is memory-based and due to the presence of COVID-19, there is a possibility of recall bias due to forgetfulness caused by the mental stress or complications of the disease and medications. Another limitation of this study was that we did not evaluate psychological history of the patients before COVID-19. Therefore, it is possible that patients with undiagnosed mental disorders were included in the study and affected the findings. However, due to the specific condition in the hospitals at the time of conducting the study, referring patients to psychologist or obtaining medical records were not possible due to the workload of the hospitals and the reduced number of functional clinics in the city.

## Conclusion

Our study showed that adherence to DASH- style diet was significantly association with depression and stress in recovered COVID-19 patients. This finding suggests that adherence to DASH-style diet can reduce depressive symptoms and mental impairments. Further longitudinal large-scale studies are necessary to establish these results.

## Data Availability

The datasets collected and/or analyzed during the present study are not publicly accessible due to ethical concerns but corresponding author may provide datasets upon reasonable request.

## Abbreviations

DASH: Dietary approaches to stop hypertension
FFQ: Food frequency questionnaire
ISI: Insomnia Severity Index
PSQI: Pittsburgh Sleep Quality Index
SARS-CoV-2: Severe Acute Respiratory Syndrome Coronavirus 2
QoL: Quality of life
DASS: Depression anxiety stress scales
